# The Association between Selective Serotonin Reuptake Inhibitors (SSRIs) Use and the Risk of Bladder Cancer: A Nationwide Population-Based Cohort Study

**DOI:** 10.3390/cancers12051184

**Published:** 2020-05-07

**Authors:** Yi-Chun Liu, Vincent Chin-Hung Chen, Mong-Liang Lu, Min-Jing Lee, Roger S. McIntyre, Amna Majeed, Yena Lee, Yi-Lung Chen

**Affiliations:** 1 Taichung Hospital, Ministry of Health and Welfare, Taichung 40343, Taiwan; purpplewhale@yahoo.com.tw; 2 School of Medicine, Chang Gung University, Tauyuan 33302, Taiwan; cch1966@gmail.com (V.C.-H.C.); 8035c@cgmh.org.tw (M.-J.L.); 3 Department of Psychiatry, Chiayi Chang Gung Memorial Hospital, Chiayi 61363, Taiwan; 4 Department of Psychiatry, Wan Fang Hospital and School of Medicine, College of Medicine, Taipei Medical University, Taipei 11696, Taiwan; mongliang@tmu.edu.tw; 5 Department of Psychiatry, University of Toronto, Toronto 399, ON M5T 2S8, Canada; roger.mcintyre@uhn.ca; 6 Mood Disorders Psychopharmacology Unit, University Health Network, Toronto 399, ON M5T 2S8, Canada; amna.majeed@mail.utoronto.ca (A.M.); yenalee.lee@mail.utoronto.ca (Y.L.); 7 Department of Healthcare Administration, Asia University, Taichung 41354, Taiwan; 8 Department of Psychology, Asia University, Taichung 41354, Taiwan

**Keywords:** antidepressants, selective serotonin reuptake inhibitor, bladder cancer, fluoxetine, Taiwan national insurance

## Abstract

Background: Past studies suggest mixed associations between selective serotonin reuptake inhibitor (SSRI) prescription and carcinogenic risk. There is no epidemiological study reporting on the association between SSRI use and the incidence of bladder cancer. The aim of this study is to determine whether SSRI use influences the risk of bladder cancer. Methods: We conducted a nationwide retrospective cohort study by Taiwan’s National Health Insurance Research Database from January 1, 1997 to December 31, 2013. 192,392 SSRI prescribed individuals were randomly matched 1 to 1 with 191,786 individuals who had never received any SSRIs by propensity scores match. The Cox Proportional Hazard models were conducted to examine the risk of bladder cancer between individuals prescribed SSRIs and individuals not prescribed SSRIs. Results: SSRIs were associated with significant reduced risk of bladder cancer with 0.5, 1, and 2 year induction periods (adjusted hazard ratio (aHR) = 0.86, 95% CI (confidence interval) = 0.76–0.98, aHR = 0.85, 95% CI = 0.75–0.97, and aHR = 0.77, 95% CI = 0.66–0.89). When examining the effect of specific SSRI, there was significantly lower risk of bladder cancer in individuals prescribed fluoxetine (6 month induction period: aHR = 0.78, 95% CI = 0.65–0.93; 1 year induction period: aHR = 0.78, 95% CI = 0.65–0.94; 2 year induction period: aHR = 0.73, 95% CI = 0.60–0.89), paroxetine (6 month induction period: aHR = 0.78, 95% CI = 0.61–0.99; 1 year induction period: aHR = 0.79, 95% CI = 0.61–1.01; 2 year induction period: aHR = 0.72, 95% CI = 0.54–0.95), and citalopram (6 month induction period: aHR = 0.74, 95% CI = 0.53–1.03; 1 year induction period: aHR = 0.70, 95% CI = 0.50–0.99; 2 year induction period: aHR = 0.60, 95% CI = 0.41–0.88). Conclusions: Individuals prescribed fluoxetine, paroxetine, or citalopram had a reduced risk of bladder cancer in this large, cross-national database.

## 1. Introduction

Bladder cancer is a highly recurrent disease [1] and is the 10th most common cancer globally [2]. Among men, it is the 9th leading cause of cancer death [2]. Based on the severity of the disease, many patients need long-term surveillance. Patients with non-muscle invasive bladder cancer need periodic cystoscopy and/or intravesical therapy. Patients with recurrent or advanced muscle invasive tumor even require radical cystectomy and adjuvant chemotherapy. These factors, amongst others, have a great impact on health-related quality of life [3].

Selective serotonin reuptake inhibitors (SSRIs) are widely used for patients with depressive and anxiety disorders as well as other therapeutic purposes. Over the past decades, there have been several studies that have evaluated the possible association between SSRIs and cancer risk. Earlier studies suggested that SSRIs increased the risk of cancer, especially breast cancer and colorectal cancer [4,5,6,7]. However, other, subsequent studies did not support these foregoing findings [8,9,10]. Moreover, an inverse relationship between SSRIs and cancer risk has also been reported suggesting an anti-cancer effect of SSRIs. For example, three population-based studies reported that SSRIs are associated with a lower risk of cancer (i.e., hepatocellular carcinoma, liver, and ovarian cancer) [11,12,13]. A separate study suggested that antidepressant prescription before bladder cancer diagnosis was associated with a lower degree of invasiveness and severity of cancer at the time of diagnosis [14]. Moreover, some animal and human cell in-vitro studies have also demonstrated the tumor suppression effect of SSRIs [15,16,17,18,19,20]. SSRI-induced increase in intracellular calcium influx in various cancer cells, including bladder, renal, oral, and prostate cancer cells was also reported in the in vitro studies. The increase in intracellular calcium levels is hypothesized to lead to apoptosis [15,16,17,18,19]. 

To our knowledge, there is no population-based study designed to survey the relationship between the use of SSRIs and incidence of bladder cancer. We conducted a nationwide population-based cohort study in Taiwan to investigate the association between SSRIs use and risk of bladder cancer. Based on extant studies [15,16,17,18,19], we hypothesized that SSRI use would further decrease the risk of bladder cancer.

## 2. Results

### 2.1. Characteristics of Subjects with or without SSRIs Use

We identified 192,392 SSRI users and 191,786 non-SSRI users in the analysis. Table 1 presents descriptive demographic, concomitant medication and comorbid illness data within SSRI users and non-SSRI users. There were 99,529 female cases (51.7%) and 92,863 male cases (48.3%) of SSRI users. After propensity score matching, there was no significant difference in sex, urbanization, medication and comorbidities between the SSRI users and non-SSRI users in terms of standardized mean difference (all standardized mean differences < 0.2). With a 6 months induction period, there were 518 cases (0.3%) with the diagnosis of bladder cancer in SSRI users and 559 cases (0.3%) in non-SSRI users. The median age of cancer diagnosis was 73 years in SSRI users, and 75 years in non-SSRIS users.

### 2.2. SSRI Use and the Risk of Bladder Cancer 

**Figure 1:**
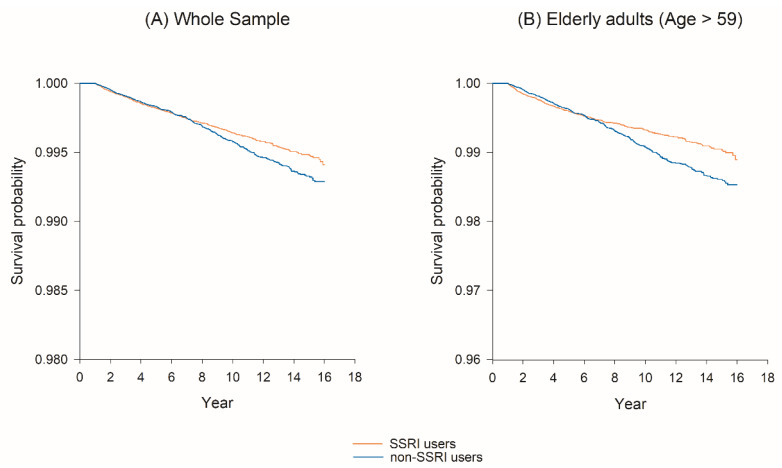
Survival probability of bladder cancer between SSRI and non-SSRI user groups.

Table 2 presents the results of the sensitivity analysis for SSRI use and bladder cancer incidence after propensity score matching for demographics, comorbidities, and medication use listed in Table 1. In the whole sample, in longer induction periods of SSRI, SSRI users had a significantly lower risk for bladder cancer with an aHR = 0.86, 95% CI = 0.76–0.98 for 6 month induction period, an aHR = 0.85, 95% CI = 0.75–0.97 for 1 year induction period, and an aHR = 0.77, 95% CI = 0.66–0.89 for 2 year induction period comparing to non-SSRI users. In the elderly adult population, there was a tendency that SSRI users had reduced risk for bladder cancer within a 6 month induction period (adjusted hazard ratio, aHR = 0.86), it did not reach statistical significance based on its 95% CI of 0.74–1.01. However, when the induction period extended to 1 year and 2 year, there existed more significant risk reduction for cancer, with an aHR = 0.83, 95% CI = 0.71–0.98, and aHR = 0.70, 95% CI = 0.58–0.85, respectively. The Kaplan–Meier survival curves presented in Figure 1 reporting the differences in survival functions of bladder cancer between SSRI and non-SSRI users. In the whole sample, SSRI users had a higher survival probability than non-users after 8 years of follow-up. Compared with the whole sample, after 8 years of follow-up, the difference in survival probabilities between SSRI users and non-users became more significant in the elderly population.

### 2.3. Specific SSRI Use and the Risk of Bladder Cancer

Table 3 presents the results of the association between specific SSRI use and the risk of bladder cancer. Sertraline comprised 42.3% (81,326 cases) of all SSRIs use, followed by fluoxetine (40.4%; 77,769 cases) and paroxetine (24.4%; 47,018 cases). Initially, the induction period applied was 6 years. After adjusting for demographics, comorbidities, and concomitant medication use within a 6 year induction period, among all SSRIs, only fluoxetine and paroxetine had significantly reduced risk for bladder cancer, with an aHR = 0.79, 95% CI = 0.66–0.95 and an aHR = 0.73, 95% CI = 0.60–0.89, respectively. When we defined the induction period as 1 and 2 years, there was prominent risk reduction for bladder cancer in fluoxetine (1 year induction, aHR = 0.78, 95% CI = 0.65–0.94; 2 year induction period, aHR = 0.73, 95% CI = 0.60–0.89), paroxetine (1 year induction, aHR = 0.79, 95% CI = 0.61–1.01; 2 year induction period, aHR = 0.72, 95% CI = 0.54–0.95), and citalopram (1 year induction, aHR = 0.70, 95% CI = 0.50–0.99; 2 year induction period, aHR = 0.60, 95% CI = 0.41–0.88) users.

## 3. Discussion

To our knowledge, this is the first population-based cohort study to assess the relationship between SSRIs use and bladder cancer risk. Our results indicate that SSRIs are associated with significantly reduced risk for bladder cancer with 6 months, 1 year, and 2 years as induction periods, by 14%, 15%, and 20%, respectively. When evaluating the SSRI individually, fluoxetine, paroxetine, and citalopram had significantly reduced risk for bladder cancer. When the induction period was defined as 2 years, after adjusting for demographic factors, concomitant medication, and other comorbid illnesses, fluoxetine, paroxetine, and citalopram reduced the risk of bladder cancer by 27%, 28%, and 40%, respectively.

The results of our study were in accordance with previous studies supporting a cancer protective effect of SSRIs. In past few years, some studies have proposed possible protective effects of SSRIs on cancer risk [11,12,13]. In Canada, Xu et al. conducted a population-based case-control study to explore SSRIs and the risk of colorectal cancer, which involved 6544 colorectal cancer cases [13]. This study reported that high daily SSRI dosage (i.e., >6.0 × 10^−6^ mol per day) before the diagnosis of colorectal cancer was associated with decreased risk of this cancer [13]. Mørch et al. also performed a similar study in Denmark to explore SSRIs and the risk of ovarian cancer. The study involved 4103 women with epithelial ovarian cancer [11]. The results revealed that SSRIs were associated with a reduced risk of epithelial ovarian cancer (OR = 0.85; 95% CI, 0.74–0.96), especially citalopram (OR = 0.78, 95% CI = 0.66–0.93) [11]. A separate Taiwanese nationwide population-based study evaluated the association between SSRIs and the risk of liver cancer [12]. It was reported that all agents within the SSRI class were associated with dose-dependent reduced risk for hepatocellular carcinoma (e.g., fluoxetine: 1-28 DDD (defined daily dose): adjusted odds ratio (aOR): 0.81, 95%CI = 0.73–0.89; 29–365 DDD: aOR = 0.71, 95% CI = 0.64–0.79; and ≥366 DDD: aOR = 0.55, 95% CI = 0.45–0.67) [12]. 

In our review, there was only one study investigating the possible associations between antidepressants (including SSRIs, SNRIs, MAOIs, and TCA) and invasive bladder cancer [14]. Steffensen et al. performed a national population-based cohort study in Denmark enclosing all cases with invasive bladder cancer during 2005–2015 [14]. The results showed that antidepressants use as a whole in the year before cancer diagnosis was associated with less advanced cancer at diagnosis (aOR = 0.86, 95% CI = 0.74–0.99) [14]. Compared to their study, our study specifically focused on the impact of all SSRIs as a group and each SSRI on bladder cancer risk. We also included all stages of bladder cancer in our analysis, instead of invasive bladder cancer only. Besides, this study did not adjust for comorbid psychiatric disorders [14]. As previous studies have shown, mood and anxiety disorder were known to have impacts on diagnosis and mortality of cancers [21,22,23,24,25]. Previous studies demonstrated that pre-existing depression or mixed depression–anxiety symptoms are associated with more advanced stage at diagnosis of cancers and poorer survival rate [21,22,23,24,25]. These findings may be due to a lower rate of health care utilization [25], relatively less receiving specialized treatment [21,24], or other common biological pathways between mood symptoms and cancers [23]. To our knowledge, SSRIs are common in the treatment of depressive and anxiety disorder. Both diseases had been reported to be associated with cancer incidence [22]. Therefore, we adjusted for these two specific psychiatric comorbidities in our analysis.

Several mechanisms had been proposed to explain the tumor suppression effects of SSRIs. One of the most mentioned mechanism is calcium-dependent apoptosis. For example, Tang et al. demonstrated that fluoxetine increased intracellular Ca^2+^ concentration via the increased release of endoplasmic reticulum Ca^2+^ storage and extracellular Ca^2+^ influx in bladder transitional carcinoma cells [15]. Other studies demonstrated not only fluoxetine but also sertraline and paroxetine could result in a similar phenomenon in different types of cancer cells [15,16,17,18,19]. A rapid rise of intracellular Ca^2+^ concentration caused mitochondria heavy burden and induced cancer cell apoptosis [17,18,19]. Moreover, an in vitro study conducted by Ahmadian et al. reported that citalopram induced apoptosis via cytochrome c release in human liver cancer (HepG2) cells [26]. According to the in vitro study by Boehning et al., cytochrome c can further sensitize inositol (1,4,5) trisphosphate receptor (InSp3R) in early apoptosis phase and lead to constant intracellular calcium increase [27]. The sustained released calcium triggers cytochrome c release, which enhances the initial apoptotic signal and further leads to cell death [27]. Besides the antitumor mechanism via apoptosis, the mechanism of the blockade of tumor cell cycle progression of fluoxetine and paroxetine had also been proposed [28,29]. For example, Lin et al. demonstrated that fluoxetine at concentrations from 5 to10 μM could activate peroxisome proliferator-activated receptor alpha (PPAR-α) and further cause G1 arrest and growth inhibition in human bladder carcinoma cell (T24) [28]. The study of Jang et al. proposed that paroxetine at 10 μM induced downregulation of the tyrosine-protein kinase Met (c-Met), and inhibited cell growth in human colon cancer cells [20]. As previous studies have reported, c-Met overexpression is common in invasive bladder cancer cells and predicted worse prognosis [30,31]. Our results supported the antitumor effects of fluoxetine, paroxetine, and citalopram on bladder cancer. However, more in vivo studies are needed to explore the specific mechanism of the antitumor effect of fluoxetine in humans.

This study is the first to utilize a representative nationwide population database cohort for exploring the effect of SSRIs on bladder cancer risk. The large sample size and complete follow-up time made our results increased generalizability. After excluding bladder cancer diagnosis prior to SSRIs exposure, the study design of ours was able to offer the possible temporal and causal relationship between them. All data including demographic information, disease diagnoses, or medical prescription came from the National Health Insurance Research Database (NHIRD) which could reduce the recall and selection bias. 

We also performed sensitivity analyses to evaluate the impact of different induction times of medication. The diagnosis of bladder cancer in our study was highly reliable based on the following reasons. The validity of cancer diagnosis in the NHI database had been confirmed with a positive predictive value of 94% for all cancers in a previous study [32]. In our study, the diagnoses recorded in the NHI database were further confirmed by utilizing the Catastrophic Illness Registry Dataset. In Taiwan, this dataset enrolls cancer patients with Catastrophic Illness Certificates. The approval of cancer patients with certificate requires diagnoses made by specialized physicians, confirmation of tissue pathology, and formal review by the Bureau of National Health Insurance.

There are several methodological limitations of our study that affect interpretations and inferences. In our study, we failed to demonstrate the dose-dependent effect of SSRIs. We had conducted the analysis to evaluate if cumulative exposure time or cumulative defined daily dose of SSRIs affect bladder cancer risk. However, the data showed no significant difference. For one reason, our study was designed as a retrospective cohort study, and randomization could not be carried out. For another, some confounding factors could not be acquired in the NHID data in this study. For example, medical adherence and stage of cancer at diagnosis could not be acknowledged. Prescription dosage and length in pharmacy records are not equal to the true exposure, which could underestimate the associations. Besides, the NHIRD did not record data about lifestyle, hair dye or perm, smoking status, diet, and occupation, which are potentially confounding factors. According to the previous review articles, cigarette smoking, occupational or industrial exposure to aromatic amines which are commonly used in hair dyes and plastic industries, and arsenic exposure in the workplace or drinking water are risk factors for bladder cancer [33,34]. By contrast, high physical activity and appropriate fruits and vegetable intake protect against bladder cancer [33,34]. 

## 4. Materials and Methods

### 4.1. Source Population

The National Health Insurance (NHI) program was initiated on March 1, 1995 by the health bureau in Taiwan. The coverage of NHI program reached 99.5% of the entire population in Taiwan in 2008, which included approximately 23 million individuals. The data analyzed in this study was derived from the data of NHI program. The National Health Insurance Research Database (NHIRD) comprised the medical claim records of hospital inpatient care, ambulatory care, and medication prescription claims data. The database de-identified these registry data and synthesized the dataset for research purposes. By utilizing a systematic sampling method, NHIRD provided a representative sample (i.e., the Longitudinal Health Insurance Database (LHID)) of the national population. The LHID comprises all the claim data of 1,000,000 people randomly sampled from all beneficiaries of the NHI program. There was no difference in gender distribution, age, or healthcare utilization between the population in the LHID and the original NHIRD [35]. The enrollee of this study was retrieved from the LHID from January 1, 1997 to December 31, 2013. The study was approved by the Ethics Institutional Review Board of Chang Gung Memorial Hospital (201901422B1). Patient-informed consent is not required as this study used existing data of the NHIRD in Taiwan which are not individually identifiable.

### 4.2. Study and Control Cohorts

This was a nationwide retrospective cohort study. As the initial study cohort, we applied for NHRID to enroll 29,548,231 participants who received at least one inpatient diagnosis of any psychiatric disorders (ICD-9 codes: 290-319) or more than two outpatient diagnoses within one year between January 1, 1997, and December 31, 2013. The exclusion criteria were those who had unknown sex status, less than 16 years old, or with any cancer diagnosis before the initial exposure of SSRIs. We further excluded those who had ever been prescribed SSRIs between 1997 and 1998 to make sure the cohort with SSRIs use was newly exposed to SSRIs and to include as many SSRIs users as possible. We defined the study cohort by any prescription of SSRIs between this study period and defined as SSRI users in our study. We operationalized the initial exposure date as index date. 

As for the comparison group, we used LHID2005 dataset. LHID2005 is composed of 1 million beneficiaries randomly sampled in the year 2005 [35]. It is a nationally representative sample. We excluded those who had ever been prescribed any SSRIs, had an unknown sex status, or whom were less than 16 years old. The comparison cohort was those without any exposure to SSRIs during 1997 to 2013. 

**Figure 2:**
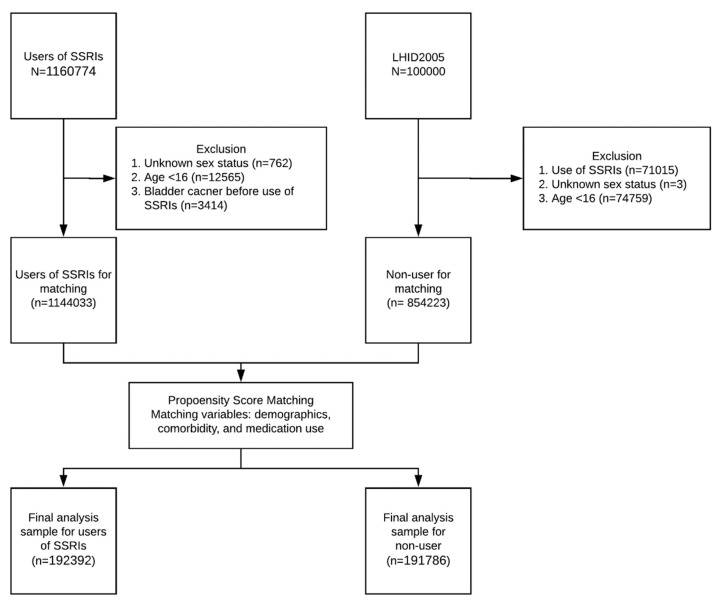
Flow chart of data collection in this study. Participants were followed from the index date, until first diagnosis of bladder cancer, death, or the end of 2013.

We used the propensity score matching to draw a comparison cohort by age, gender, comorbidity, medication, and index date. We matched subjects on the logit of the propensity score using a caliper of a width of 0.001. The follow-up period began from the initial exposure of SSRIs (the index date) and terminated when the first date of bladder cancer diagnosis was made, at death, or at the end of 2013. The bladder cancer diagnosis was defined by at least one inpatient diagnosis or twice outpatient diagnoses. The diagnosis was recorded in the International Classification of Diseases, Ninth Revision (ICD-9) code of 188, and confirmed by using the Catastrophic Illness Registry Dataset. In Taiwan, the diagnosis of bladder cancer is made by specialized urologists and based on urine cytology with malignant cells or pathology study with a biopsy sample through cystoscopy. The flow chart of participants selection in this study was presented in Figure 2. The validity of bladder cancer diagnosis is reliable in NHIRD as shown in a previous study [32]. In a previous study, Kao et al. compared all cancers diagnoses from the NHIRD to those from the National Cancer Registry in Taiwan [28]. The positive predictive value of cancer diagnoses for all cancers in the NHIRD was 94% [28]. To avoid immortal time bias, SSRI users and non-SSRI users were followed up after the induction period of initial SSRI use (the index date) and from the matched dates of starting follow-up, respectively. Patients who developed bladder cancer before the follow-up index dates were excluded at the data washing period.

### 4.3. SSRI Exposure 

Following the Anatomical Therapeutic Chemical (ATC) code, the SSRIs included in our study were N06AB05 (paroxetine, Whole Win Pharmaceutical Co., Ltd., Taipei, Taiwan), N06AB03 (fluoxetine, Taiwan Biotech Co., Ltd, Taoyuan, Taiwan), N06CA03 (fluoxetine and psycholeptics, Standard Chem & Pharm Co., Ltd, Tainan, Taiwan), N06AB06 (sertraline, Nang Kuang Pharmaceutical Co., Ltd, Tainan, Taiwan), N06AB10 (escitalopram, EB Pharmaceutical Ltd, Taipei, Taiwan), N06AB04 (citalopram, Shou Chan Industrial Co., Ltd, Nantou, Taiwan), and N06AB08 (fluvoxamine, Taiwan Biotech Co., Ltd, Taoyuan, Taiwan). For causal relationship, we defined the empirical induction period as 6 months [36]. Subsequently, we conducted sensitivity analyses to evaluate a range of plausible empirical induction periods (i.e., one year and two years). 

### 4.4. Data Analyses

Descriptive statistics of SSRIs exposure and comparison cohorts were reported, including demographic characteristics, healthcare system utilization, comorbid illness, and exposure to potentially confounding drugs. Urbanization levels were stratified into urban and rural areas [37]. Comorbid illnesses included in the analysis were as follows: anxiety disorder (ICD-9-CM code 300), depressive disorder (ICD-9-CM code: 300.4, 311, 296.2, 296.3), diabetes mellitus (ICD-9-CM code 250), hypertension (ICD-9-CM code: 401, 997.91), hyperlipidemia (ICD-9-CM code: 272.2, 272.4), alcohol use disorder (ICD-9-CM code 305.0, 305.00, 305.01, 305.02, 305.03), tobacco use disorder (ICD-9-CM code 305.1), obesity (ICD-9-CM code: 279.0, 278.01, 278.00), Chronic Obstructive Pulmonary Disease (COPD) including bronchitis, not specified as acute or chronic (ICD-9-CM code 490), chronic bronchitis (ICD-9-CM code 491), emphysema (ICD-9-CM code 492), and chronic airway obstruction, not elsewhere classified (ICD-9-CM code 496), chronic cystitis (ICD-9-CM code 595.1, 595.2), polycystic kidney (ICD-9-CM code V18.61, 753.12, 753.13, 753.14), and calculus of kidney and ureter (ICD-9-CM code 592.0, 592.1, 592.9). Five drugs included for adjustment were aspirin (ATC code: B01AC06), non-steroidal anti-inflammatory drugs (NSAIDs) (ATC code: M01A), statins (ATC code: C10AA), cyclophosphamide (ATC code: L01AA01), and pioglitazone (ATC code: A10BG03).

Standardized mean differences were used to evaluate the difference of matching variables between the SSRI user group and the non-SSRI user group. A value of 0.2 or greater of the standardized mean difference indicated a notable difference between the two groups [38]. We used the robust Cox proportional hazard model to take propensity score matching strata into account and to assess the risk of bladder cancer according to each category of antidepressant exposure status. After controlling for demographics, comorbidities, and medication mentioned above, we calculated the adjusted hazard ratios (HRs) and 95% confidence intervals (Cls) for bladder cancer. A Kaplan–Meier survival curve was used to present the differences of survival function between SSRI users group and the non-SSRI users group. Because bladder cancer is more prevalent in older people, especially for those more aged than 60 years past, we subgrouped two populations to perform sensitivity analysis [39]. One was the whole population group, and the other was the group aged 60 years or older. Sensitivity analyses were also conducted to evaluate the association with different induction periods (six months, one year, and two years) of SSRIs use and bladder cancer. Statistical significance was set at 0.05. All analyses were conducted using the SAS Version 9.4 software (SAS Institute, Cary, NC, USA)

## 5. Conclusions

Our study found the protective effect of fluoxetine, paroxetine, and citalopram on bladder cancer. Whether protective effect of fluoxetine, paroxetine, and citalopram on bladder cancer suffices clinical application depends on further human or molecular studies.

## Figures and Tables

**Table 1:** Comparison of demographic characteristics and medical diseases in patients with or without selective serotonin reuptake inhibitors (SSRIs) after propensity score matching.

Variable	Users of SSRIs	Non-Users of SSRIs	Standardized Mean Difference
	N = 192,392	N = 191,786	
**Sex**			
Men	92,863 (48.3%)	91,032 (47.5%)	−0.0075
Women	99,529 (51.7%)	100,754 (52.5%)	
**Age (years)**			
≤50	88,914 (46.2%)	75,981 (39.6%)	−a
51–60	31,781 (16.5%)	33,197 (17.3%)	
61–70	21,356 (11.1%)	24,239 (12.6%)	
>70	50,341 (26.2%)	58,369 (30.4%)	
**Urbanization**			
Urban	158,421 (82.3%)	156,864 (81.8%)	−0.006
Rural	33,971 (17.7%)	34,922 (18.2%)	
**Associated disease**			
Anxiety disorder	76,704 (39.9%)	83,066 (43.3%)	0.035
Depressive disorder	18,812 (9.8%)	14,701 (7.7%)	−0.021
Diabetes mellitus	35,256 (18.3%)	40,644 (21.2%)	0.029
Hypertension	67,205 (34.9%)	77,025 (40.2%)	0.053
Hyperlipidemia	15,486 (8.0%)	18,259 (9.5%)	0.015
Alcohol use disorder	726 (0.4%)	693 (0.4%)	<0.001
Tobacco use disorder	2791 (1.5%)	2698 (1.4%)	<0.001
Obesity	1221 (0.6%)	1225 (0.6%)	<0.001
COPD	32,328 (16.8%)	36,134 (18.8%)	0.021
Chronic cystitis	1184 (0.6%)	1188 (0.6%)	<0.001
Polycystic kidney	172 (0.1%) 180 (0.1%) 172 (0.1%)	180 (0.1%)	<0.001
Calculus of kidney and ureter	67,975 (35.3%)	6873 (3.6%)	0.003
**Medication use**			
Aspirin	67,975 (35.3%)	72,363 (37.7%)	−0.016
NSAIDs	93,151 (48.4%)	89,883 (46.9%)	0.005
Statins	19,735 (10.3%)	20,544 (10.7%)	0.024
Cyclophosphamide	1069 (0.6%)	1237 (0.6%)	0.001
Pioglitazone	5542 (2.9%)	5887 (3.1%)	0.002
**Outcome**			
Bladder cancer	518 (0.3%)	559 (0.3%)	-
Age of diagnosis, Median (IQR)	73 (44–89)	75 (51–87)	

Standardized mean difference is not available for multicategorical variable. COPD = Chronic Obstructive Pulmonary Disease. IQR = interquartile range.

**Table 2:** Sensitivity analysis for SSRI use and bladder cancer incidence in different induction periods after propensity score matching.

Induction Period	Adjusted HR (95% CI) ^a^	
	Whole Sample	Elderly Adults (Age > 59)
	N = 384178	N = 154305
6 months	0.86 (0.76–0.98) *	0.86 (0.74–1.01)
1 year	0.85 (0.75–0.97) *	0.83 (0.71–0.98) *
2 years	0.77 (0.66–0.89) **	0.70 (0.58–0.85) ***

^a^ Analyses were adjusted for demographics, comorbidity, and medication use listed in Table 1. * *p* < 0.05, ** *p* < 0.01, *** *p* < 0.001.

**Table 3:** Association of SSRI use and the risk of bladder cancer.

Specific SSRIs	N (%)	6 Months	1 Year Induction Period	2 Year Induction Period
		Adjusted HR (95%CI)	Adjusted HR (95%CI)	Adjusted HR (95%CI)
Fluoxetine	77,769 (40.4)	0.78 (0.65–0.93) *	0.78 (0.65–0.94) *	0.73 (0.60–0.89) *
Paroxetine	47,018 (24.4)	0.78 (0.61–0.99) *	0.79 (0.61–1.01)	0.72 (0.54–0.95) *
Sertraline	81,326 (42.3)	1.03 (0.74–1.43)	1.00 (0.70–1.43)	1.00 (0.66–1.51)
Escitalopram	40,740 (21.2)	1.03 (0.74–1.43)	1.00 (0.70–1.43)	1.00 (0.66–1.51)
Citalopram	25,971 (13.5)	0.74 (0.53–1.03)	0.70 (0.50–0.99) *	0.60 (0.41–0.88) **
Fluvoxamine	16,403 (8.5)	0.94 (0.65–1.37)	1.04 (0.70–1.55)	1.06 (0.68–1.65)

Users of specific SSRIs were compared to their matched control using propensity score in Table 1. Analyses were conducted using a 1 year induction period and a 2 year induction period and adjusted for demographics, comorbidity, and medication use listed in Table 1. * *p* < 0.05, ** *p* < 0.01.
